# The effect of robot speed on comfortable passing distances

**DOI:** 10.3389/frobt.2022.915972

**Published:** 2022-07-26

**Authors:** Margot M. E. Neggers, Raymond H. Cuijpers, Peter A. M. Ruijten, Wijnand A. IJsselsteijn

**Affiliations:** Human-Technology Interaction, Eindhoven University of Technology, Eindhoven, Netherlands

**Keywords:** proxemics, human-aware robot navigation, personal space, human comfort, passing distance

## Abstract

Robots navigate ever more often in close proximity to people. In the current work, we focused on two distinctive navigational scenarios: passing and overtaking a person who is walking. In the first experiment, we compared nine different passing distances for a humanoid robot and found that human comfort increased with passing distance and that their relationship could be described by an inverted Gaussian. In the second experiment, we validated this relationship for an industrial autonomous robot and extended the study to also include overtaking distances and different robot moving speeds. The results showed that overtaking was considered to be less comfortable than passing but that the overtaking distance had a similar relationship with human comfort. Human comfort decreases with a higher robot movement speed. Results obtained through location trackers furthermore showed that people actively take a larger distance from the robot when it starts its trajectory closer to them. The current results can be used to quantify human comfort in environments where humans and robots co-exist and they can be used as input for human-aware navigational models for autonomous robots.

## 1 General introduction

Robots are increasingly often navigating in close proximity to people. This holds for social robots such as care robots (Breazeal, 2011), but also for industrial robots such as robots operating on farms (Heidari and Fotouhi, 2014). It is desirable that people accept the robots and get used to them co-existing in the same environments. We argue that if the behavior of the robot is implemented such that it adheres to human social rules for navigation, it is more likely that people are able to understand the robot’s behavior. For example, it is necessary that robots respect social distances (Althaus et al., 2004).

**FIGURE 1:**
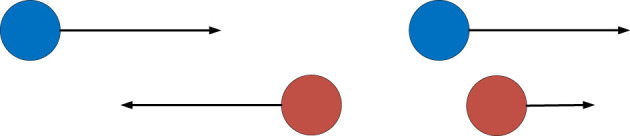
Schematic overview of the two scenarios: on the left passing and on the right overtaking.

In the current paper, we describe two distinct situations that can frequently occur when robots and humans are navigating in the same environment: passing and overtaking. Passing is defined as when two actors are starting their trajectory opposite from each other and pass each other along the way. Overtaking is defined as when two actors start their trajectory at the same side the faster one overtakes the other actor. See Figure 1 for a schematic overview of the two scenarios.

These two scenarios need to be implemented in the behavior of the robot in a socially aware and natural manner, such that robots are more likely accepted navigating in human environments. We start with investigating the human social rules of navigation and social distances.

### 1.1 Personal space and social navigation

Hall introduced personal space in his book “The Hidden Dimension” (Hall, 1966). He referred to the study of social distances as proxemics. Interactions of different types require different distances between individuals, which is why Hall decided to define different zones of personal space. Intimate interactions such as embracing or whispering take place in the intimate zone ( < 0.45 m), while for a regular conversation it is more appropriate to take a distance according to the social zone (around 1.2 m). When in interaction with another human, people subconsciously take these distances into account. Otherwise, their interaction partner could feel uncomfortable or awkward. The distances that belong to the zones are dependent on cultural preferences. On the shape of this personal space is still some discussion, Hall considered it to be circular (Hall, 1966), but other researchers found egg-shaped (Beck and Ollendick, 1976; Bailenson et al., 2001), elliptical (Helbing and Molnar, 1995) or asymmetrical (Gérin-Lajoie et al., 2008) shapes of personal space. For a more detailed overview of these shapes you can read the work by Rios-Martinez et al. (2015) or by Neggers et al. (2022).

For most of these shapes of personal space, people are considered to be static. However, it is hypothesized that personal space may be different when people are moving. Indeed, people require more space towards their front when they are moving. Kitazawa and Fujiyama (2010) found that moving pedestrians use a so-called information-processing space, which extends roughly 4.5 m ahead of the pedestrian. Within this space, they detect possible collision risks, such that they can avoid them in time. This assumption is also made by Corbetta et al. (2018). They furthermore found that moving pedestrians aim to keep a distance of 80 cm from other pedestrians when they pass by.

It is still not clear whether the same rules of human social navigation apply to robots. In our earlier study (Neggers et al., 2022), we found that a passing distance of 80 cm was indeed considered comfortable, but in this study, people were not moving but stationary.

### 1.2 Human-aware robot navigation

Gao and Huang (2021) summarized different evaluation methods and metrics for human-aware navigation. Often it starts with a case study, in which a specific navigational scenario is described. Another method could be simulating or analyzing existing data sets of navigational behaviors. The third method can be defined as lab studies, where certain metrics such as human (dis)comfort or robot navigational performance are studied by exposing human participants to controlled robot behaviors. Often the last method employed is to test the results or complete navigational algorithms on a real robot in the field.

There have been a lot of simulation studies on robots passing and overtaking people (Chen et al., 2017; Vega et al., 2019; Manso et al., 2020). However, for lab studies on robot proxemics, the focus is often on comfortable stopping distances when a robot is approaching a user to start a social interaction, such as a conversation (Walters et al., 2009; Torta et al., 2013; Mead and Matarić, 2016; Rossi et al., 2017). The results of these studies differ. In some studies it is found that robots need to keep a larger distance than humans usually do (Torta et al., 2013), even well out of the personal zone as defined by Hall (1966). In other studies, results show that robots are allowed to come closer (Walters et al., 2009), in the intimate zone as defined by Hall (1966). It appears to depend on the age, needs, and personality of the user, the skills and appearance of the robot and the overall context of the situation Koay et al. (2017).

Much fewer lab studies aim to quantify personal space in a dynamic context (Pacchierotti et al., 2006; Neggers et al., 2022). More often human-aware robot behavior in navigation is tested in a complete navigational model, such as the Human-Aware Motion Planner by Sisbot et al. (2007). In this model, people are represented by cost maps. These cost maps allow the robot to always keep a safe distance from a human. They furthermore prevent the person from being startled by the robot, by taking a larger passing distance when the robot passes a person at their back, or when the robot emerges from behind a wall. This is mainly applicable for a static person, when a person is moving the cost map should extend towards the front (Kirby et al., 2009). However, to be able to form these cost maps, the constraints first need to be quantified.

### 1.3 Passing, overtaking and moving speed

There are almost no studies that focus on quantifying human comfort and robot efficiency for passing or overtaking scenarios. In our own earlier work (Neggers et al., 2022), we investigated passing distances for a static person. We found that human comfort is a nice smooth function of passing distance and that passing at the back of the person was perceived as less comfortable compared to passing at their sides or at the front. The cost map we generated based on the experimental results was similar to the Human-Aware Motion Planner (Sisbot et al., 2007), which is also used for static people. They also considered static humans to be more comfortable with larger passing distances of the robot and put a higher cost for robots passing at a person’s back. A study that tested different passing distances for moving people was the work by Pacchierotti et al. (2006). A robot passed a human in a hallway and used different passing distances. They found that larger passing distances again resulted in higher human comfort.

As for overtaking, to our knowledge, there are no studies that systematically compare comfortable overtaking distances. There are navigational models that take overtaking into account, but they usually follow a field study approach (Pandey and Alami, 2010). This means that they do not base their assumptions on real-life data.

In most studies on navigational models, only one movement speed is employed. Robot movement speeds and the effect they have on comfortable passing distances are not often compared. One exception is the study by Zhang et al. (2019). They found that the moving speed of a (virtual) robot had an effect on the distance people kept between them and the robot. The larger the movement speed, the bigger the distance. This could indicate that people are feeling less safe or comfortable with higher moving speeds of the robot. Clearly, higher speeds involve more risk of a collision and more potential damage than low speeds, so it makes sense that people feel this way.

### 1.4 Research aims

The main aim of the paper is to investigate the relationship between passing distances of robots and the experiences comfort of humans. We present two studies with specified research goals.

In Experiment 1 we aim to experimentally find the most comfortable passing distance when a robot passes a person who is walking forward down a corridor. The experiment can give us more insight into how a passing distance affects the perceived comfort of a moving person. We expect people to feel more comfortable with increasing passing distances based on Pacchierotti et al. (2006), however, it is not yet clear how comfort should be quantified as a function of distance. We furthermore expect based on Bitgood and Dukes (2006) that people are more comfortable with a robot passing on their left side because that is coherent with traffic rules in most EU countries, including the Netherlands, where this study was executed. We test these expectations in the first study in a controlled experiment where we manipulate the passing distance and side between a human and robot and evaluate perceived comfort.

In Experiment 2 we aim to determine how the moving speed of the robot affects the relationship between passing distance and comfort. Additionally, we aim to compare the effect of passing (starting at opposite sides of the corridor) and overtaking (starting at the same side of the corridor) on perceived comfort. We expect based on Zhang et al. (2019) that perceived comfort is lower for higher moving speeds of the robot. We test these expectations in a controlled experiment where we manipulate passing distance and side, moving speed, and scenario, and we evaluate perceived comfort.

## 2 Experiment 1: passing distances

### 2.1 Method

#### 2.1.1 Participants and design

Thirty-two participants (17 males and 15 females, *M*_*age*_ = 32.0, *SD*_*age*_ = 16.3, range = 19–81) participated in the first experiment. The participants were sampled from the participant database of the Eindhoven University of Technology. This database consists mainly of students at the same university but also working or retired people from the region are registered. Participants indicated little below average familiarity with robot on a 7-point Likert Scale (*M*_*familiarity*_ = 3.47, *SD*_*familiarity*_ = 1.65). For the majority, the familiarity with robots consists of participating in other experiments.

All participants participated in a within-subjects design with 9 (minimal passing distance: 50–130 cm in steps of 10 cm) × 2 (passing side: left or right) × 2 (sequence: descending or ascending order) trials. Passing side alternated between each trial, and the passing distance would either increase or decrease with steps of 10 cm after each trial, depending on the sequence. Each participant experienced each of the 36 trials in the same order. Due to technical issues with the robot, 72 out of the 1152 trials could not be used (6.25%).

#### 2.1.2 Materials and measurements

**FIGURE 2:**
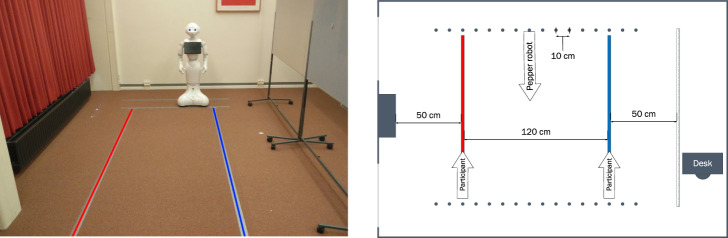
Overview of the experimental setup. In the left part of the figure, the experiment room, temporary walls, and the robot are shown. Participants walked on the red and blue lines. The robot had different starting positions as indicated by the dots together with a schematic overview of the dimensions in the right part of the figure. Reproduced from Figure 1 in Neggers et al. (2018).

The Pepper robot (Softbank Robotics, Japan) was used in the experiment. In the experiment room, a corridor was created with a width of 220 cm using poster boards, as can be seen in Figure 2. The length of the corridor was about 4 m. On the floor two lines of tape (one red and one blue) were applied, to indicate where the participant should walk. The Pepper robot started at one of the indicated starting positions opposite of the participant and drove with a speed of 0.35 m/s along a straight line. The schematic overview of the setup can be found in Figure 2. There were no social interactions between the robot and the participant, the robot did not change its gaze heading and did not use any gestures.

After each trial participants were asked to rate their agreement with two statements on a 7-point Likert scale: “I felt comfortable passing the robot” and “The robot passed me at a comfortable distance”. These questions were highly correlated with each other with *r*(30) = 0.86, and *p* < 0.001, so the average value of the two was used in the analysis.

#### 2.1.3 Procedure

Participants arrived at the lab where they were informed about the procedure of the experiment, and signed an informed consent form. They first completed a few demographic questions on their age, gender, and familiarity with robots. Afterward, the experiment started. The computer on the desk indicated in Figure 2 showed them in which direction (passing the temporary wall on the left or right side) and on which line (red or blue) they were supposed to walk. The robot started at the opposite side and passed the participant at a constant speed. After each trial, the participant filled in the two questions on comfort followed by a new walking instruction on the computer. This procedure was repeated for all 36 trials in a fixed order. The first trial was at a distance of 50 cm with the robot on the left side of the participant. Left and right would alternate between trials and the distance would increase for the first half of the experiment, after which it decreased again. The total experiment lasted 30 min for which participants were paid €5.

### 2.2 Results

#### 2.2.1 Comparing comfort values

**FIGURE 3:**
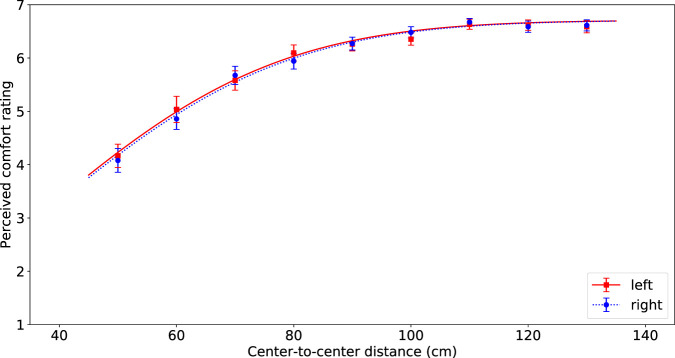
Visualization of the average comfort level per passing distance for the robot passing on the left (red squares) and right (blue circles) of the participant. The error bars represent Standard Error. The lines indicate the best fitting inverted Gaussian, solid red line for left and dashed blue line for right.

**FIGURE 4:**
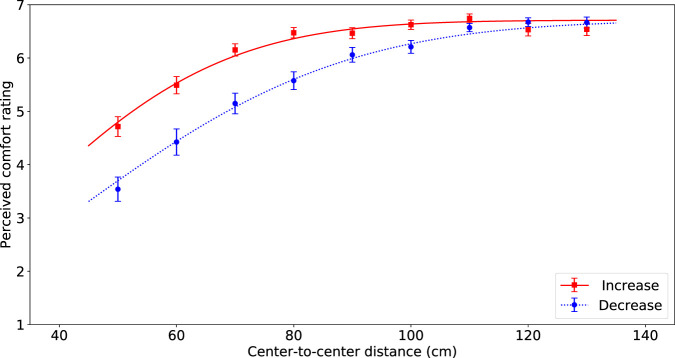
Visualization of the average comfort level per passing distance for the passing distances of the robot increasing (red squares) and decreasing (blue circles) of the participant. The error bars represent Standard Error. The lines indicate the best fitting inverted Gaussian, solid red line for increase and dashed blue line for decrease.

To test the effects of passing distance, passing side, and sequence on comfort, we conducted a repeated measures Analysis of Variance (rANOVA). The average values of comfort across the different conditions are visualized in Figures 3, 4.

The ANOVA indicated that the different passing distances had a significant effect on the comfort values (*F*(8, 66) = 128.71, *p* < 0.0001, *η* ^2^ = 0.50). Perceived comfort was higher for larger passing distances. The lowest comfort ratings were used in evaluation of a distance of 50 cm (*M* = 4.12, SD = 1.75) and the highest comfort ratings were for a distance of 110 cm (*M* = 6.65, SD = 0.64). Pairwise comparisons with a Bonferroni correction for multiple tests indicated that from a distance of 90 cm the subsequent pairs of distances do not differ significantly from each other, but up until that distance, the subsequent pairs do differ significantly.

Additionally, we found a significant effect of sequence on perceived comfort (*F*(1, 66) = 119.37, *p* < 0.0001, *η* ^2^ = 0.11). If the distance of subsequent trials followed a decreasing order the mean on comfort was lower (*M* = 5.64, SD = 1.63) compared to when distances followed an increasing order (*M* = 6.19, SD = 1.44).

There was no significant effect of passing side on perceived comfort (*F*(1, 66) = 0.03, *p* = 0.86). Eye-balling Figure 3 shows that there are almost no differences between the bar heights for left and for right passing sides, despite one instance for a passing distance of 60 cm.

Finally, results showed an interaction effect between sequence and distance on the perceived comfort values *F*(8, 66) = 11.26, *p* < 0.0001, *η* ^2^ = 0.08). In Figure 4 it is clear to see that the effect of distance is bigger for the decreasing sequence. No other interaction effects were found to be significant.

#### 2.2.2 Finding an appropriate fit

In order to evaluate the effects found and model them, we fitted a non-linear model. A second-order polynomial seemed a good fit. However, there was no clear indication of the comfort values dropping again beyond our measured range. Even though the average comfort values of a distance of 120 and 130 cm were lower than the value of 100 cm, these differences were not significant. Therefore it seems safer to assume that the comfort values saturate for larger passing distances. Additionally, using a second-order polynomial means that negative comfort ratings could occur beyond the measured rate, both for small and for large passing distances.

Another possibility for fitting the data was an exponential function. This formula provided a good fit since the comfort ratings would saturate to a maximum value with large distances. However, this only works if the intercept is unrealistically small. This means that the formula also gives negative comfort ratings for small distances.

A formula that does not suffer from these drawbacks is the inverted Gaussian. This function as given by Eq. 1 goes to 0 when the minimal passing distance x approaches 0 and approaches a maximum comfort level for large minimal passing distances. An additional benefit is that it requires one parameter less than the other two proposed functions.

$$g\left(x\right)=1+a_{0}\left[1-\exp\left(-\frac{x^{2}}{2\sigma^{2}}\right)\right]$$ (1)

#### 2.2.3 Fitting comfort values

We used the formula on the inverted Gaussian to find the best fit for our data. We submitted the data to a non-linear regression using passing distance, passing side, and sequence as predictors of comfort. We used Eq. 2 as shown below:

$$C\left(d\right)=1+a_{0}\left[1-\exp\left(-\frac{d^{2}}{2\left(\sigma^{2}+a_{1}\epsilon +a_{2}\eta \right)}\right)\right],$$ (2)

in which *C* represents the perceived level of comfort, *d* represents the minimum passing distance and *ϵ* and *η* are dummy variables. *ϵ* has the value of 1 if the robot passed on the right and 0 if the robot passed on the left. Similarly, *η* is 1 if the passing distances are increasing with trial number and 0 if the distances are decreasing with trial number. *σ* is the width of the inverted Gaussian, *a*_0_ is the height of the inverted Gaussian, and the variables *a*_1_, *a*_2_ represent the effect size of passing side and whether distance was increasing or decreasing. The results of the non-linear regression are shown in Table 1.

**TABLE 1:** Fitted parameter values and summary statistics of Eq. 2.

Parameter	Estimate	SE	*t*-value	*p*
*σ*	44.0	1.04	42.3	< 0.0001
*a*_0_	5.71	0.06	101.1	< 0.0001
*a*_1_	0.51	1.02	0.50	0.617
*a*_2_	−10.4	1.02	−10.2	< 0.0001

Same as in the ANOVA, no significant effect of passing side was found. We plotted the fitted functions on the data in Figure 3, and as the figure clearly shows, the two lines are almost identical. Therefore we decided to set this effect to zero, which changes the other parameters slightly, see Table 2.

**TABLE 2:** Fitted parameter values and summary statistics of Eq. 2, with the effect of passing side (*ϵ*) set to zero (*a*_1_ = 0).

Parameter	Estimate	SE	*t*-value	*p*
*σ*	44.2	0.90	48.06	< 0.0001
*a*_0_	5.71	0.06	101.2	< 0.0001
*a*_2_	−10.4	1.02	−10.2	< 0.0001

The effect of sequence was significant, as is clearly shown in Figure 4. When the robot used increasing passing distances the perceived comfort level was higher (especially for the smaller distances), compared to when the robot used decreasing passing distances.

### 2.3 Discussion

The aim of this first experiment was to find a comfortable passing distance for a robot when passing a person in a corridor. Our main finding is that perceived comfort increases with passing distance following an inverted Gaussian. In our other work, the same relationship is found (Neggers et al., 2022), and as we show in that paper, the same relationship can be found in the work of Pacchierotti et al. (2006). This gives further proof that an inverted Gaussian is an appropriate way of describing the relationship between passing distance and perceived comfort.

Clearly, there are many possible functions that have a similar sigmoid shape like the inverse logit function or the arctangent function. With sufficient parameters, they all are able to describe our data. However, the inverted Gaussian is better in the sense that it gives theoretically sound predictions for very large and very small distances. In addition, it provides a very good fit with only 2 parameters. The first parameter (*a*_0_) determines the response range of participants and reflects details of the experimental method. The second parameter (*σ*) determines the steepness of the curve and is therefore directly related to how people take the social distance into account.

Our results show that there is a gradual transition from low to high comfort, so it is difficult to use these results to set an acceptable passing distance. It is possible to take an arbitrary value for perceived comfort, e.g., 6, and use Eq. 2 to check which passing distance corresponds with this comfort value. For example, a comfort level of 6 would result in a center-to-center distance of 80 cm, which is an edge-to-edge distance of approximately 36 cm. This value corresponds with a human passing distance for pedestrians, as found by Corbetta et al. (2018).

Although there is some agreement with other literature, an acceptable comfort rating of 6 was chosen arbitrarily. We expect that in practice what people find acceptable will depend on many variables as was for instance shown by Heerink et al. (2010). They identified 13 variables that all affect user acceptance of social robots. For some people it might be necessary to reach a higher comfort level, e.g., elderly people might prefer a larger distance (Khaksar et al., 2021). Alternatively, in specific contexts, it might be necessary to make a trade-off between robot efficiency and human comfort, and there developers might choose to take a lower perceived comfort criterion.

In the current data we found no support of an effect of passing side, although we did expect some difference due to the traffic rules (e.g., driving on the right side of the road). We believe that this effect is not present because of the context of the experiment. In some situations, task constraints are more important than the social convention (Kirby et al., 2009). In the current study, people were instructed to walk over a line, which is a clear task constraint. If the robot and human would start on a collision course and the robot would actively choose a side that does not correspond with the social convention of passing at each other’s right, it might be considered as uncomfortable.

Finally, we found a significant effect of sequence on perceived comfort. This effect is clearly visible as a hysteresis effect in Figure 4. Outside the lab passing distances would never occur in perfect sequences, which makes sequence less meaningful in practice. The effect that we found can probably be explained by the fixed questionnaire. Participants may have compared their current comfort level with their comfort level during the previous trial. This would explain why a decreasing sequence (the robot coming closer each trial) leads to a lower comfort level overall than an increasing sequence where the robot is moving further away after each trial. One could speculate that some form of adaptation is taking place, but we have no evidence to support this notion. It might be that people dislike it when a robot is coming closer during the passing, however, future research is necessary to investigate this effect further.

## 3 Experiment 2: Passing distances at various speeds

In the second experiment, we used an industrial autonomous robot instead of a humanoid robot. Next to the passing distance and side, we varied the moving speed of the robot and the scenario (passing or overtaking).

### 3.1 Method

#### 3.1.1 Participants and design

Twenty-two participants (11 males and 11 females, *M*_*age*_ = 23.9, *SD*_*age*_ = 3.42, range = 19–35) participated in the second experiment. The participants were again sampled from the participant database of the Eindhoven University of Technology. Two participants indicated encountering robots on a weekly basis, four participants see them on a monthly basis, eleven participants on a yearly basis and three participants indicated never encountering robots in their daily life. The participants who indicated a frequent encounter explained this mainly as participating in other robot experiments or having industrial robots in their workplace. The average walking speed of the participants was 1.00 m/s ± 0.15 m/s. This is a bit slower than regular human walking speed (Fitzpatrick et al., 2006), but suitable for the corridor length and the accompanying starting and stopping period.

All participants participated in a within-subjects design with 4 speeds (0.35, 0.8, 1.25 or 1.7 m/s) × 2 sides (left or right) × 4 distances (60, 80, 100 or 120 cm) × 2 scenarios (overtaking or passing). The overtaking scenarios were only coupled with the two highest speeds (1.25 and 1.7 m/s) which resulted in 48 unique trials. These 48 trials were presented in a random order, but there was a certain structure to keep the time of the experiment short. Left and right were alternated, such that the robot and the participant just had to turn around in order to be ready for the next trial. There were at most 3 switches between the two scenarios because to switch scenario the participant had to walk to the other side of the room with the robot staying in the same position. The speeds and distances were fully random.

For one participant the order of the trials was mixed up during the experiment, which meant that there were duplicate trials. The data of this participant is not taken into account in the analysis. The data of the other 21 participants could all be used.

#### 3.1.2 Materials and measurements

**FIGURE 5:**
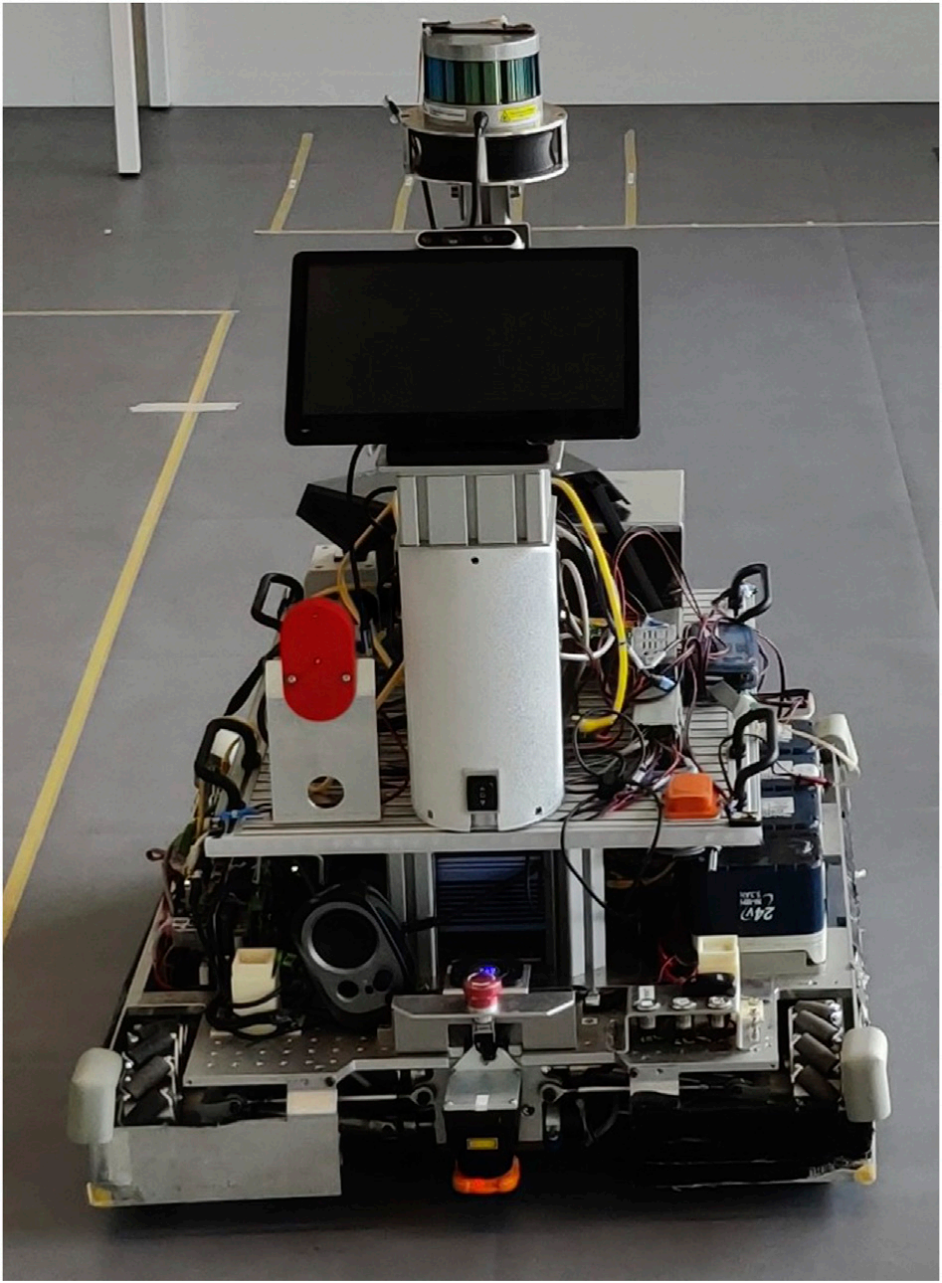
Custom built AGV used in the second experiment.

**FIGURE 6:**
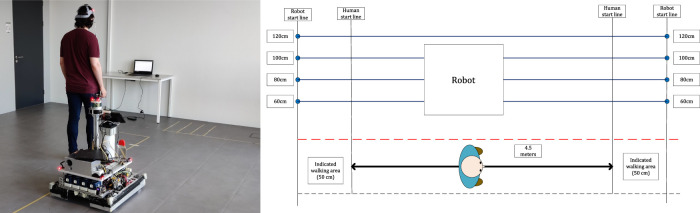
Overview of the experimental setup. On the left side of the experiment room, the participant and robot are shown. One of the laptops where people could fill in the questionnaire is visible on the picture. The robot had different starting positions as indicated by the circles in the sub-figure on the right side with the passing distances of the robot indicated next to them.

For this experiment, we used a custom-made Autonomous Guided Vehicle (AGV), as shown in Figure 5. This robot has dimensions of L: 425 mm, W: 480 mm, H: 1210 mm. In a lab room (6 × 6m) we indicated the starting position of the robot and the participant, as indicated in Figure 6. The robot only drove on one side of the lab room, and we used tape to indicate the red line in the Figure. Participants were not allowed to cross this line, to guarantee their safety. The length of the walking path for participants was 4.5 m.

After each trial participants were asked to answer one question on a 7-point Likert Scale: “How comfortable were you with the passing of the robot?”

Additionally, we used location trackers of PhaseSpace Motion Capture, to track the location of the participant with respect to the location of the robot. We used this data to analyze whether people actively took more distance from the robot than indicated.

#### 3.1.3 Procedure

Participants arrived in the lab. They disinfected their hands according to the COVID-protocol, that was in place at the time. They were instructed about the procedure of the experiment, after which they signed the informed consent form. They had to wear a headband with the LEDs for the location tracker and they put this on themselves, after which the experiment started. They walked from one side of the room to the other side and the robot would either start from the same side or the other side, depending on the scenario (passing or overtaking). After walking the prescribed trajectory, participants went to laptop and answered a question about their perceived comfort. They experienced all 48 trials in a semi-random order. In the end, they answered a few demographic questions. The experiment lasted 45 min, for which participants received €7.50.

### 3.2 Results

#### 3.2.1 Comparing comfort values

First, we established whether there was an effect of robot speed, passing distance, and side and scenario type on comfort. Therefore, we conducted a full-factorial Analysis of Variance (ANOVA).

**FIGURE 7:**
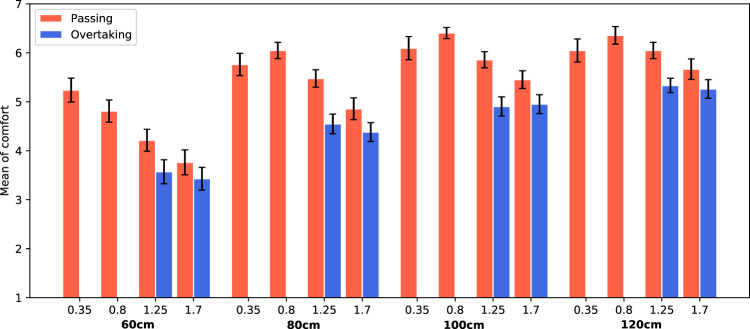
Average comfort values for both trials. The passing side (left/right) is averaged. Error bars represent Standard Error.

The ANOVA indicated a significant main effect of passing distance on comfort (*F*(3, 940) = 110.23, *p* < 0.0001, *η* ^2^ = 0.26). Further analysis shows that the larger the distance between the robot and the participant, the higher the comfort values. Another significant main effect was found for scenario (*F*(1, 940) = 64.00, *p* < 0.0001, *η* ^2^ = 0.06). Participants were less comfortable for the overtaking scenario (*M* = 4.55, SE = 0.25), compared to the passing scenario (*M* = 5.51, SE = 0.06). The speed of the robot also showed to have a significant main effect on the perceived comfort (*F*(3, 940) = 30.20, *p* < 0.0001, *η* ^2^ = 0.09). Higher speeds are less comfortable. In Figure 7 the average comfort levels are shown for each condition. Clearly, the average comfort levels are higher for larger passing distances. The comfort values for overtaking (blue columns) are always lower than for passing (red columns). Finally, for passing scenarios, the comfort levels decrease with increasing speed.

**FIGURE 8:**
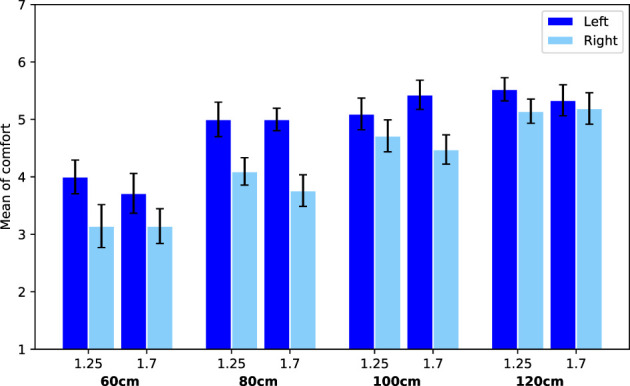
Average comfort values for the trials with scenario overtaking. Error bars represent Standard Error.

**FIGURE 9:**
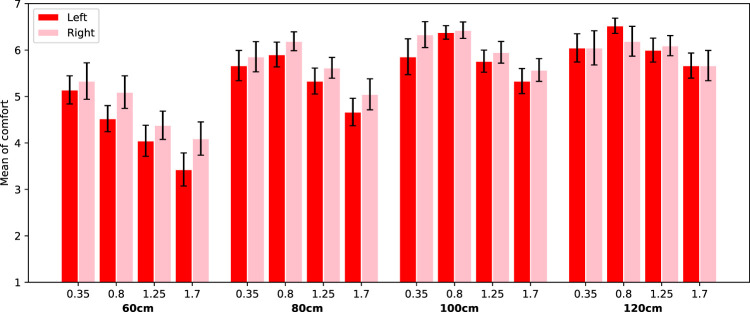
Average comfort values for the trials with scenario passing. Error bars represent Standard Error.

Additionally, a main effect for passing side was found (*F*(1, 940) = 7.07, *p* = 0.008, *η* ^2^ = 0.01). This effect is further specified by a significant interaction effect between passing side and scenario (*F*(1, 940) = 37.87, *p* < 0.0001, *η* ^2^ = 0.04). Looking into this effect, we see that when the robot is overtaking, people are less comfortable when this happens at their right (*M* = 4.21, SE = 0.11) compared to their left side (*M* = 4.89, SE = 0.10). However, when the robot is passing, people are less comfortable with the robot at their left (*M* = 5.39, SE = 0.08) compared to their right side (*M* = 5.62, SE = 0.08). See Figure 8 and Figure 9, where this effect is clearly visible.

Another significant interaction effect was found between the robot speed and the scenario (*F*(1, 940) = 6.06, *p* = 0.01, *η* ^2^ = 0.006). Because not all speeds were tested in both scenarios, it is difficult to interpret this interaction effect. However, Figure 7 shows that the effect for speed is more present for the passing scenario than for the overtaking scenario, as the bars of the latter scenario are roughly on the same level for both speeds that were tested.

#### 3.2.2 Fitting comfort values

After comparing the comfort values we fitted an inverted Gaussian for the different scenarios. We tried to include speed as a parameter in the same way as we did for passing side and sequence in the first study, however, this did not give good results. Therefore we fitted each speed and passing scenario separately with an inverted Gaussian, as presented below:

$$c\left(d\right)=1+a_{0}\left[1-\exp\left(-\frac{d^{2}}{2\sigma^{2}}\right)\right],$$ (3)

**FIGURE 10:**
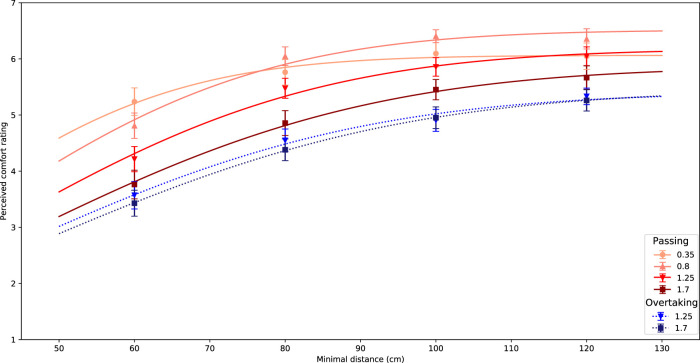
Fitted inverted Gaussians for all combinations of scenarios and speed. Error bars represent Standard Error.

The results are shown in Figure 10. For each scenario and speed, the inverted Gaussian proved to be a good fit. Eye-balling this graph shows us that the two functions for overtaking (dotted lines) are both lower than the functions for passing (continuous lines) and that there is not much difference in the two functions for overtaking, showing that moving speed of the robot did not have much effect in this scenario. However, comparing the four functions for passing behavior show that overall a higher moving speed gives a higher comfort level, but the lowest moving speed of 0.35 m/s appears to be the odd one out, as it flattens for higher passing distances. For small passing distances (60 cm) this curve still shows the highest comfort levels, but for large passing distances ( > 80 cm) the moving speed of 0.8 m/s gives higher comfort levels.

**FIGURE 11:**
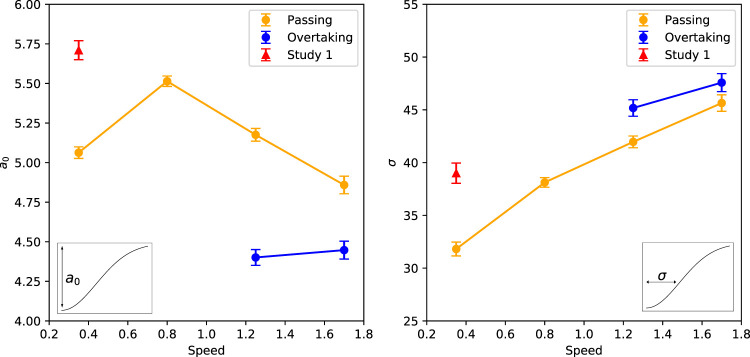
Parameters *a*_0_ and *σ* plotted with the movement speed of the robot. *a*_0_ represents the height and *σ* represents the width of the inverted Gaussian describing the relationship between comfort and passing distance (see insets). In the figure the parameters of Experiment 1 (red triangles) and Experiment 2 for both passing (yellow circles) and overtaking (blue circles) are plotted. Error bars represent Standard Error.

To further quantify our results, we take a closer look at the parameters. The inverted Gaussian as shown in Eq. 3 has two fixed parameters: *a*_0_ depicting the height of the Gaussian and *σ* depicting the width of the Gaussian. We plotted the parameters against the speed of the robot in Figure 11. This gives some insight into Figure 10. Overtaking shows a lower overall comfort compared to passing scenarios, which is shown by both speeds with a smaller height of the Gaussian, indicated by a lower *a*_0_. The width of the Gaussian is comparable between the two scenarios.

We also use Figure 11 to explain flattening of the curve of a movement speed of 0.35 m/s. Looking at the *a*_0_ parameter in the left panel, we see a negative linear relationship between the three highest movement speeds (0.8, 1.25 and 1.7 m/s) in the passing scenario. This is confirmed with a linear regression analysis on these three speeds ( $R_{\mathrm{adj}}^{2}$ = 0.9996, *p* = 0.012). However, clearly the movement speed of 0.35 m/s does not fall on the same line. If we include the *a*_0_ parameter of a movement speed of 0.35 m/s, the relationship is no longer significant ( $R_{\mathrm{adj}}^{2}$ = 0.2003, *p* = 0.55). This means there are two options, either the relationship between *a*_0_ and robot movement speed is not linear, or the speed of 0.35 m/s is an outlier. We plotted the *a*_0_ parameter of the first experiment in the same graph in red and with a triangle shape, and this one does appear to follow the same negative linear relation as the other three. A linear regression indeed indicates a significant relation, if we replace the *a*_0_ parameter of Experiment 2 with the one for Experiment 1 ( $R_{\mathrm{adj}}^{2}$ = 0.9823, *p* = 0.006). This could mean that the measurement of a movement speed of 0.35 m/s was an outlier in the second experiment.

However, looking at the right panel of Figure 11 that shows the *σ* parameters, all four movement speeds of Experiment 2 seem to follow a positive linear relationship which is confirmed by a linear regression analysis ( $R_{\mathrm{adj}}^{2}$ = 0.9715, *p* = 0.010). In this case, replacing the *σ* parameter of movement speed of 0.35 m/s with the one from experiment 1 shows an insignificant linear relationship ( $R_{\mathrm{adj}}^{2}$ = 0.7236, *p* = 0.097). So for this parameter it appears as if the sigma parameter of Experiment 1 is the outlier which could indicate that this can be explained by a difference between the two experiments. The movement speeds in Experiment 2 show a clear relation with each other. More data is necessary to define which of the two explanations hold.

#### 3.2.3 Analysing path data

**FIGURE 12:**
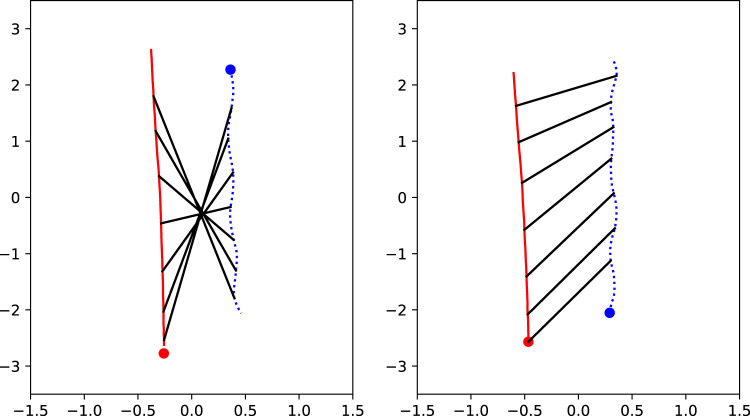
Examples of location data. The red solid line represents the path of the robot, with the dot as starting location. The blue dotted line represents the path of the participant, with the dot as starting location. The black lines represent the relative location of the actors with respect to each other. The numbers on the axes represent meters. On the left side, a passing scenario is shown with a passing distance of 60 cm and a robot moving speed of 1.7 m/s, and on the right side, an overtaking scenario is shown with a distance of 80 cm and a robot moving speed of 1.7 m/s.

We also tracked the location of the participant and the robot with a frequency of 10 Hz. We used this data to recreate the walking trajectory of the participant with respect to the robot. Examples are shown in Figure 12.

The graphs show that the path of the robot veers towards its left side. This is probably a control issue with one of the wheels, and it was not noticed during the experiment. For the interpretation of the conditions, it means that when the robot starts at the participants’ left side it is converging toward the participant in its path, while when the robot starts at the participants’ right side it is diverging away from the participant. We took a closer look at the path data and found that the average difference between the beginning and end position of the robot was 15 cm. On a total travelled distance of 5 m for the robot, this is not a large deviation. It slightly changes the actual passing distances and shifts the curves in Figure 10 a bit, but it would not change the effect of our manipulations.

Next we used the location data for a new parameter. We calculated the closest distance between the robot and the participant during the trial. Because this distance is related to our manipulated starting distance, we subtracted the starting distance from the minimum distance to correct for the starting position, to create a new variable, which we call path deviation. Because the unanticipated deviation of the robot as explained in the previous paragraph was minimal, we expect that the value of this path deviation to reflect participants’ preferred distances to the robot. If participants would not deviate at all from their starting position this value would be close to 0. If they would deviate from their path away from the robot the value would be positive. If they would deviate towards the robot the value would be negative. In some trials the location trackers were not working properly, so for these trials, we could not calculate the distance. This was the case for 11 out of 1008 trials (1.1%).

To estimate the effect of our independent variables on the path deviation we conducted a full factorial Analysis of Variance (ANOVA).

The ANOVA indicates a significant main effect of scenario on path deviation (*F*(1, 929) = 151.80, *p* < 0.0001, *η* ^2^ = 0.14). The path deviation was clearly higher for overtaking scenarios (*M* = 15.76 cm, SE = 1.33 cm) than for passing scenarios (*M* = 3.65 cm, SE = 0.34 cm). Upon investigating the right panel of Figure 12, it is clear that in some cases the robot was not successful in overtaking the participant, and kept a little bit behind the participant. This is shown by the angle of the black lines. If the robot would succeed in overtaking the participant they would be horizontal somewhere in the middle and after that point they would flip over. In this case, you can see that the angle never becomes horizontal, meaning that the robot was always behind the participant. Especially with a moving speed of 1.25 m/s, the robot did not often succeed in overtaking the participant. This can explain the large differences in path deviations between scenarios, as for passing there was always a moment where the robot and the participant were at the same level.

This is further confirmed by a significant interaction effect between moving speed and scenario (*F*(1, 929) = 38.95, *p* < 0.0001, *η* ^2^ = 0.04). For the overtaking scenario there is a large difference between the path deviation for a moving speed of 1.25 m/s (*M* = 21.77 cm, SE = 2.22 cm) and a moving speed of 1.7 m/s (*M* = 9.68 cm, SE = 1.31 cm). For the passing scenario there are no significant differences for the different robot moving speeds.

The ANOVA showed a significant main effect of the manipulated distance on the path deviation (*F*(3, 929) = 13.41, *p* < 0.0001, *η* ^2^ = 0.04). The path deviation for a manipulated distance of 60 cm was higher (*M* = 11.68 cm, SE = 1.12 cm) than for the manipulated distances of 80, 100, and 120 cm, with 80 cm being the highest of these three (*M* = 7.55 cm, SE = 1.07 cm). A small but significant interaction effect between scenario and distance (*F*(3, 929) = 3.32, *p* = 0.019, *η* ^2^ = 0.01) indicates that this effect holds for both scenarios but with larger differences for each of the manipulated distance levels for the overtaking scenario. This is probably a subconscious reaction of our participants related to comfort: people take a larger distance to the robot when the initial positions are closer together.

Another significant main effect is the effect of passing side (*F*(1, 929) = 35.64, *p* < 0.0001, *η* ^2^ = 0.04). Further inspection shows a significant interaction effect between passing side and scenario on path deviation (*F*(1, 929) = 113.06, *p* < 0.0001, *η* ^2^ = 0.11). This confirms our impression that the robot deviated towards the left during the experiment. In the overtaking scenario the robot starting at the left of the participant gave higher path deviations (*M* = 22.79 cm, SE = 1.66 cm) than the robot starting at the right (*M* = 8.82 cm, SE = 1.94 cm). For the passing scenario this effect is opposite, there were higher path deviations when the robot started at the right side of the participant (*M* = 5.25 cm, SE = 0.44 cm) compared to when it started at the left side of the participant (*M* = 2.04 cm, SE = 0.50 cm).

Lastly, we checked whether the participant deviation significantly impacted the comfort ratings given by participants by including the variable as a covariate into our full factorial ANOVA. Their was no significant effect of participant deviation on the given comfort ratings (*F*(1, 881) = 0.18, *p* = 0.67), nor did including the factor in our model change the earlier interpretations of the results.

The location trackers also allowed us to estimate the average walking speed of a participant during a trial. With a full factorial ANOVA we checked for significant effects of our manipulations on the average walking speed of the participants. We found a significant main effect of robot speed on average walking speed (*F*(3, 931) = 26.11, *p* < 0.0001, *η* ^2^ = 0.07). A pairwise comparison shows that if the robot had a moving speed of 0.35 m/s the average walking speed of our participant was significantly lower (*M* = 0.94 m/s, SE = 0.02 m/s) compared to a moving speed of 0.8 m/s (*M* = 1.00 m/s, SE = 0.01 m/s), 1.25 m/s (*M* = 1.00 m/s, SE = 0.01 m/s) or 1.7 m/s (*M* = 1.01 m/s, SE = 0.01 m/s).

### 3.3 Discussion

The results of the second study show a clear effect between passing distance and perceived comfort. The relationship between these two variables can again be described by an inverted Gaussian. An interesting addition to the second study is the location tracking of our participants, that is consistent with the comfort ratings. In this case, the walking behavior of the participants also clearly shows an effect of passing distance. For the smallest passing distance, the difference between the manipulated passing distance and measured path deviation was greater than for the other passing distances. This shows that people actively evade the robot when it is very close.

Currently, the only actor that could change its path during the passing or overtaking was the person. However, in a human-human collision avoidance scenario, it is common to share the effort of avoiding a collision (Olivier et al., 2013). It might be that people get annoyed if the robot does not actively diverge from a straight path when both actors are starting close together. Furthermore, according to Lu and Smart (2013), diverging from a straight path is also a cue indicating the robot has noticed a person and takes them into account. Further research should investigate the effect of a robot actively changing its path on human comfort.

We see a clear effect of scenario: passing is considered to be more comfortable than overtaking. This can probably be explained by the predictability of the robot’s behavior. In our earlier work (Neggers et al., 2022), we also found that a robot passing at a person’s back is considered to be more uncomfortable than passing a person’s front. At the front, a person can use vision, to predict where the robot is going. At the back, a person has to depend on their auditory senses to predict the current and future location of the robot, which is presumably less precise.

The current results also confirm that the inverted Gaussian is a good fit for the relationship between passing distance and perceived comfort. The fit is a good predictor of the data in both scenarios and all moving speeds. Only the parameters *σ* and *a*_0_ change across the different conditions. The current study gives some insight into how the moving speeds affect these parameters. Clearly, the moving speed of the robot has effect on the perceived comfort of the human. In general, it means for higher speeds that the inverted Gaussian is less high and wider. However, the slowest moving speed deviated from this pattern. It furthermore deviates from the results of the first experiment, where the same movement speed was used.

We consider two possible explanations for this result. In the experimental set-up of the second experiment, the participant had to wait for the robot to finish its trajectory before the next trial would start. We saw in the location data that the participants already compensated for this by walking slower themselves, as indicated by slower average walking speeds, but it still often happened during the experiment that participants already filled in the questionnaire for the previous trial and afterward spent some time waiting for the robot to arrive at its destination. This might have caused awkward situations, which might have affected participants’ comfort levels. Furthermore, they could easily compare this with the other trials in which the robot and the participant would arrive roughly at the same time. In the first experiment people would fill in the questionnaire behind a poster board, so they would be less confronted with the robot still arriving at its destination. Additionally, in Experiment 1, there was only one moving speed, so the waiting time between trials did not vary much. Another explanation might be that the slow moving speed of the robot was perceived differently by the participants. Knowing that the robot could drive much faster from other trials, it might have been unclear why the robot was driving so slow in comparison to their own walking speed. This might have caused uncomfortable feelings. It is hard to say which of the two explanations is best. Further research is necessary to understand why we found these results.

Contrary to the first study, we found an effect of passing side. Participants showed higher comfort values when the robot passed them at their right compared to at their left. For overtaking the effect was reversed. There are multiple possible reasons for this discrepancy, and without further research it is difficult to say with certainty which is the correct explanation. We discuss the two most likely explanations. The first possible explanation is that the traffic rules might be responsible for this effect (Bitgood and Dukes, 2006). However, we did not find support for that in Experiment 1. In Experiment 2, it could only explain the effect we find for overtaking. In the Netherlands, traffic overtakes only on the left side, meaning that when a car overtakes on the right, people are uncomfortable. However, for passing it is not according to the traffic rules, because passing also usually happens on the left side, and in the current experiment we found this to be slightly more uncomfortable. The second possible explanation of the difference between the two experiments is that the analysis of the location tracking data in the second experiment shows us that, albeit minimal, the robot diverged towards the left. This means that when it overtakes a person at their right or passes them at their left they are coming closer towards the end. We might have found an effect here of diverging away from a person, or towards a person, which is according to expectations, because diverging away is more comfortable than towards a person. Further research is necessary to find out how much a robot should diverge. However, the initial idea is interesting, and matches with the idea that a robot should share effort in avoiding collision by diverging away from the person (Silva and Fraichard, 2017).

## 4 Limitations and future research

Any experiment of this kind will be limited by the number of variables that can reasonably tested in a single study, and the potential combinations and permutations of such variables. Moreover, the choice of physical environment in which tests are performed will also offer constraints. For example, in both experiments the length of the corridor was short. In the second experiment this lead to the robot not always being able to surpass the participants in the overtaking scenario, because of the necessary time to accelerate and decelerate in the short corridor. However, the scenarios were still different enough, because for the passing scenarios the robot would start at the front of the participant and for the overtaking scenario it would start at their back. This makes the collected data and the comparison still valuable. Furthermore, we could use location trackers because we opted to perform the experiment in a lab, and this resulted in valuable behavioral data. Yet, in future research it is good to replicate the findings in a longer corridor.

In both studies presented in this work, the robots did not react to the behavior of the participant. This was done to be able to test exact passing and overtaking distances. In the real world, the robot probably needs to actively react to the behavior of the person, by for example choosing a passing side and passing distance. An added benefit of this is that the robot likely also acknowledges the presence of the person with the change in behavior (Lu and Smart, 2013). Future research should investigate what the effect of a change in the robot’s behavior is on the relationship between passing distance and human comfort.

Finally, the next step would be to validate the current results in a real-life environment, where people are unaware of the experimental set-up. A controlled lab experiment as we presented here is a good method for comparing different robot behaviors, but participants are more aware that they are being observed. Validating the current results in a field study can provide more insight into desirable robot behaviors.

## 5 General conclusion

Robots are increasingly navigating in human environments. In the current study, we aimed to investigate the effect of passing distance and robot moving speed on perceived comfort. We furthermore compared two different types of robots in the two experiments. The results of the two experiments are similar, which bodes well for the generalisability of the results and its associated model to other potential robots and situations. It also resonates with the findings of our previously published work on the topic (Neggers et al., 2022). For other robots and situations the parameters as used in the inverted Gaussian do need to be quantified, but based on the current results we contend that the relationship between passing distance and perceived comfort is similar as in the current measured situations.

To conclude, the current results show a clear effect of passing distance on perceived comfort, measured both subjectively and through behavior. The effect can be modeled using an inverted Gaussian, which only depends on two parameters. The moving speed of the robot affects these parameters, showing lower comfort levels overall for higher moving speeds. People are furthermore less comfortable with the robot overtaking them, than with the robot passing them. The current results can be used to design human-aware robot navigation models that actively monitor human comfort levels. This can make robots navigating in human environments more socially aware.

## Data Availability

The raw data supporting the conclusions of this article will be made available by the authors, without undue reservation.
